# Appendicitis Inflammatory Response Score in Comparison to Alvarado Score in Acute Appendicitis

**DOI:** 10.1055/s-0041-1731446

**Published:** 2021-07-19

**Authors:** Toney Jose, PS Rajesh

**Affiliations:** 1 Department of Surgical Gastroenterology, Bangalore Medical College and Research Institute, Bangalore, India; 2 Department of General Surgery, Government Medical College, Kottayam, Kerala, India

**Keywords:** appendicitis, clinical prediction rule, Alvarado, appendicitis inflammatory response score

## Abstract

Appendicitis is a common differential diagnosis of right lower quadrant pain. Clinical evaluation alone results in high negative appendicectomy rates. Alvarado scoring is the most commonly used clinical prediction rule. The study aimed to compare the recently developed appendicitis inflammatory response (AIR) score with the Alvarado score. This cross-sectional observational study included patients who underwent appendicectomy for clinical suspicion of appendicitis. The clinical and laboratory parameters required for obtaining Alvarado score and AIRS were gathered. Area under ROC curve was calculated for both Alvarado score and AIRS. The study included 130 patients (77 males and 53 females). The negative appendicectomy rate was 10.7%. The perforation rate was 10.3%. The area under ROC for Alvarado score was 0.821 and for AIR score was 0.901. The Alvarado score had a sensitivity of 72% and a specificity of 79% at score ≥6. The appendicitis inflammatory response score had a sensitivity of 98% for scores ≥5 and a specificity of 97% for score ≥6. The C-reactive protein (CRP) value was the best performing individual parameter with an area under ROC of 0.789, followed by WBC count with an area under ROC of 0.762. Appendicitis inflammatory response score is a recently developed score that outperforms the Alvarado score. AIR score has a higher specificity. The sound construction, gradation of parameters, the inclusion of CRP, and avoidance of subjective parameters make the AIR score an attractive clinical prediction rule which can decrease the rate of negative appendicectomy.

With a lifetime incidence of 7%, acute appendicitis is a common clinical condition, one of the many differential diagnoses of a patient presenting with acute right iliac fossa pain. 1 Many of the patients presenting to surgical emergency with acute right iliac fossa pain, have alternative pathologies. A negative appendicectomy rate up to 17.5% is noted in India following appendicectomy based on clinical suspicion. 2 This necessitates accurate diagnosis prior to performing appendicectomy. Controversies exist regarding the management of equivocal cases, with some advocating early surgical intervention and others opting for active observation. 3 4 5 Many clinical and imaging tools have been developed to aid in the diagnosis of acute appendicitis. Clinical scoring systems like appendicitis inflammatory response (AIR) score, 6 Alvarado, 7 RIPASA, pediatric appendicitis score, etc, with varying sensitivity and specificity. Imaging modalities like ultrasonography and computer tomography are also used to evaluate for inflamed appendix. 8

Even though the Alvarado score is the most extensively evaluated in validation studies, it has some drawbacks. 9 10 It was developed by retrospective analysis of patients who underwent appendicectomy for suspected appendicitis and the dichotomization of variables has led to decreased discriminative ability. AIR score was constructed from a prospective cohort, with the grading of variables for greater discrimination, lesser reliance on symptoms, and more on clinical signs. 7 It also includes C-reactive protein (CRP) as a component with graded scoring. CRP is found to be well correlated with acute appendicitis in many studies. 11 12 AIR score assigned patients to a high probability zone with substantially higher specificity (97%) and positive predictive value (88%) than the Alvarado score (76 and 65%, respectively). 13

The use of scoring systems is imperative in a resource-limited setting like India. It is useful in the clinical diagnosis, early referral from primary health care facilities, and for deciding on surgical intervention especially when imaging is inconclusive or not available. The study objective was to compare the performance of appendicitis inflammatory response score and Alvarado score among patients who underwent appendicectomy for suspected acute appendicitis.

## Methods

This cross-sectional observational study was performed in the Department of General Surgery of Government Medical College, Kottayam from January 2017 to June 2018 after obtaining ethical clearance from the Institutional Review Board. Based on sample size calculation the minimum sample size was estimated to be 122. Consecutive patients aged 12 to 60 years, who were referred to the General Surgical team on-call with acute right iliac fossa pain and were operated on for suspected acute appendicitis during the study period and gave consent for the study were included. Patients without documented signs, symptoms, and biochemical parameters required for calculating AIR score and Alvarado score, those without histopathology report of the resected specimen, and those with chronic abdominal pain were excluded from the study. The decision to operate was taken by a senior surgical staff member. Variables that were recorded included presence of nausea, vomiting, anorexia, migration of pain to the right lower quadrant (RLQ), pain in the RLQ, tenderness in RLQ, rebound tenderness or muscular defense, body temperature, white blood cell (WBC) count, proportion of polymorphonuclear leukocytes, and CRP. Alvarado score included migration of pain (score—1), anorexia (score—1), nausea or vomiting (score—1), tenderness in RLQ (score—2), rebound pain (score—1), elevated temperature (score—1), leukocytosis (score—2), and shift to left (score—1). 7 AIR score included vomiting (score—1), pain in RLQ (score—1), rebound tenderness or muscular defense (light—score 1; medium—score 2; strong—score 3), temperature (>38.5°C—score 1), polymorphonuclear leukocytes (70–80%: score 1; ≥85%: score 2), WBC count (10.0–14.9 × 10 ^3^ /mcl: score 1; ≥15.0 × 10 ^3^ /mcl: score 2), and CRP (1–4.9 mg/L: score 1; ≥5 mg/L: score 2). 6 The diagnosis was confirmed by histopathology in all resected specimens. Appendicitis was pathologically diagnosed when infiltration of the muscularis propria by neutrophil granulocytes was seen. For the purpose of the study, all histologically confirmed cases were considered to have appendicitis. Statistical analysis was performed using SPSS statistical software 23 (SPSS Inc, Chicago, IL). Continuous variables are described as mean ( ± SD). Comparison of continuous variables was done by independent-samples *t-* tests. Diagnostic accuracy was analyzed using receiver operating characteristic (ROC) curves. Statistical significance was attributed at the 5% level.

## Results

From January 2017 to June 2018, 130 patients (77 males and 53 females) who underwent appendicectomy for suspected acute appendicitis and met the inclusion and exclusion criteria were included in the study. The mean age was 27.5 ± 13.3 (range 12–70). 14 patients (six females and eight males) had normal appendix in histology giving a negative appendicectomy rate of 10.7%. The mean Alvarado score among patients with acute appendicitis in histology was 7.4 ± 1.42 as compared with 5.6 ± 1.08 among patients with normal appendix ( *p* < 0.001). The AIR scores were 7.3 ± 2.23 and 4.21 ± 0.97 ( *p* < 0.001), respectively. Thirteen patients (10.3%) had a perforated appendix at surgery. The mean Alvarado score was 7.25 for nonperforated appendix and 8.46 for perforated appendix ( *p* = 0.004). The mean AIR score for nonperforated cases was 6.99 and 10.23 for perforated cases ( *p* <0.001.)

**Fig. 1:**
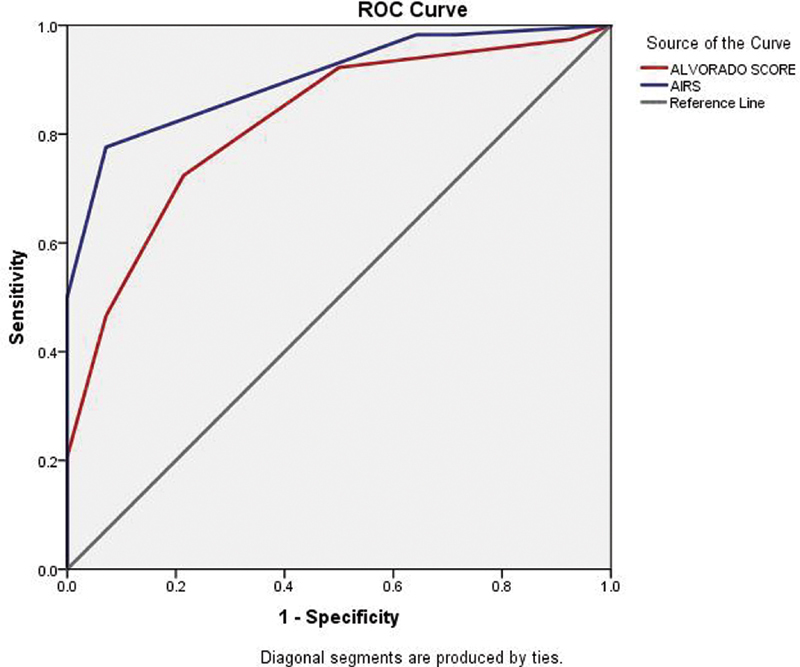
The combined ROC curves for Alvarado score and AIR score. The area under ROC for AIR score is 0.901 and the area under ROC for Alvarado score is 0.821. AIR score outperforms Alvarado score in predicting acute appendicitis. AIR, appendicitis inflammatory response; ROC, receiver operating characteristic.

The area under the ROC curve was 0.901 for the AIR score as compared with 0.821 for the Alvarado score ( Fig. 1 ). The Alvarado score has a sensitivity of 72% and a specificity of 79% at a score ≥6. For score ≥7, the sensitivity dropped to 46% whereas the specificity reached 93%. The AIR score showed a sensitivity of 98% for scores ≥5 with a specificity of 36%. The score ≥6 AIR score had a sensitivity of 77% with a specificity of 97%.

**Fig. 2:**
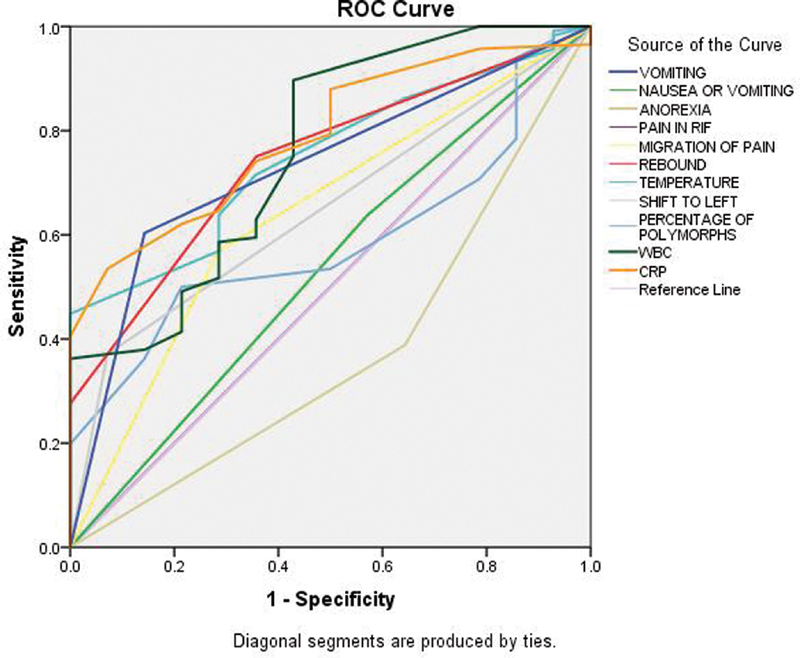
The ROC curves for individual parameters. The areas under ROC are—vomiting (0.73), nausea or vomiting (0.533), anorexia (0.373), pain in right iliac fossa (0.50), migration of pain (0.642), rebound tenderness (0.744), temperature (0.75), shift to left (0.650), percentage of polymorphs (0.589), white blood cell count (0.762), and C-reactive protein (0.789). The best performing among the individual parameters was the CRP and the worst performing was anorexia. CRP, C-reactive protein; ROC, receiver operating characteristic.

The ROC curves for the individual parameters are shown in Fig. 2 . Vomiting alone has a better area under ROC than nausea and vomiting combined (0.73 vs. 0.53). CRP value was the best performing with an area under ROC of 0.789, followed by WBC count with an area under ROC of 0.762. The poorest performing among symptoms and signs was anorexia with an area under ROC of 0.37 and the poorest performing laboratory investigation was shifted to left with an area under ROC of 0.65. CRP of ≥10.5 had a sensitivity of 74.1% and specificity of 64.3%, whereas a CRP ≥11.5 had a sensitivity of 64.7% and a specificity of 71.4%.

## Discussion

Appendicectomy is often the first major surgery to be undertaken by a surgeon in training. The clinical findings supplemented by investigations help in confirming the clinical suspicion. The use of radiological imaging has resulted in a decrease in the rates of negative appendicectomies. However, the imaging facilities are not accessible to all, especially in rural areas and, also during the night hours—when radiology facilities are not available in many hospitals. Also, imaging can be inconclusive at times. This makes the physician rely on the various clinical prediction scores.

Flum et al analyzed the data from the Washington State Database and identified 63,707 patients who underwent appendicectomy and they have noted a negative appendicectomy rate of 15.5%. 14 Sharma et al noted a negative appendicectomy rate of 23.72%, which was 13.43% in males and 37.25% in females. 2 Güller et al in an analysis based on the prospective database of the Swiss Association of Laparoscopic and Thoracoscopic Surgery (SALTS) which included patients aged 12 years and over undergoing emergency laparoscopic appendicectomy between 1995 and 2006 noted a negative appendicectomy rate of 6.5% and a perforation rate of 16.5%. 15 In our study of 130 patients, the negative appendicectomy rate was noted to be 10.7%. The mean age of the patients who had negative appendicectomy was 19.9. This was in contrast to the mean age of patients with appendicitis in histopathology which was 28.4 ( *p* = 0.005). This indicates the lower threshold in operating on young individuals as compared with the elderly.

AIR score was developed by Andersson and Andersson using the prospective data of 545 patients with suspected acute appendicitis. Comparing the AIR score and Alvarado score, the authors reported an area under ROC of 0.97 versus 0.92 ( *p* = 0.0027) for patients with advanced appendicitis and 0.93 versus 0.88 ( *p* = 0.0007) for all patients with appendicitis. Sixty-three percent of the patients were classified into the low- or high-probability group with an accuracy of 97.2%, leaving 37% in intermediate probability for further investigation. Seventy-three percent of the nonappendicitis patients were correctly classified into low probability. Sixty-seven percent of the advanced appendicitis patients and 37% of all appendicitis patients were correctly classified into the high-probability zone. 6 De Castro et al among 941 consecutive patients with suspected acute appendicitis noted that the area under the ROC curve of the AIR score was significantly better than that for the Alvarado score (0.96 vs. 0.82, *p* < 0.05). The AIR score also outperformed the Alvarado score in more difficult patients, including women, children, and the elderly. 16

Kollár et al evaluated the AIR score and compared its performance in predicting risk of appendicitis to both the Alvarado score and the clinical impression of a senior surgeon. The three methods of assessment stratified similar proportions (approximately 40%) of patients to a low probability of appendicitis ( *p* = 0.233). The false-negative rate was <8% and did not differ between the AIR score, Alvarado score, or clinical assessment. The Alvarado score had a higher specificity and positive predictive value than the Alvarado score (97 and 88% vs. 76 and 65%) in assigning patients to high probability zone. 13

Comparison between AIR score, Alvarado score, and Pediatric Appendicitis score was done by Macco et al in a retrospective study on 747 children (<18 years). It was noted that the area under the receiver-operating curve for the AIRS, Alvarado score, and the Pediatric Appendicitis score was 0.90, 0.87, and 0.82, respectively ( *p* < 0.05). In children with a low-risk acute appendicitis, false-negative rates of 14, 7, and 18% were seen for the AIR score, Alvarado score, and the Pediatric Appendicitis score. 17 In a randomized trial on AIR score-based management of patients with suspected appendicitis, among low-risk patients, the use of the AIR score-based algorithm resulted in less imaging (19·2 vs. 34·5%; *p* < 0·001), fewer admissions (29·5 vs. 42·8%; *p* < 0·001), fewer negative explorations (1·6 vs. 3·2%; *p* = 0·030), and fewer operations for nonperforated appendicitis (6·8 vs. 9·7%; *p* = 0·034). 18

In this study, the area under ROC for Alvarado score was 0.821 and for AIR score was 0.901. The Alvarado score showed better sensitivity whereas the AIR score showed better specificity. The Alvarado score has a sensitivity of 72% and a specificity of 79% at a score ≥6. When considering score ≥7, the sensitivity dropped to 46% whereas the specificity reached 93%. The AIR score had a sensitivity of 98% for scores ≥5 with a specificity of 36%. Score ≥6 showed a sensitivity drop to 77% with a specificity of 97%. Scott et al noted a high sensitivity (90%) for AIR score of 5 or more (intermediate and high risk) among all severities of acute appendicitis which increased to 98% for advanced appendicitis. Ninety-seven percent specificity was noted for a score of 9 or more (high risk) with 70% among them having perforation or gangrene. 19

In a meta-analysis by Frountzas et al RIPASA score was found to have an area under the curve of 0.94 as compared with the Alvarado score with 0.79. 20 Chisthi et al noted that the Modified Alvarado score had an area under the curve of 0.72 as compared with 0.94 for the AIR score and 0.91 for the RIPASA score. 21 In their study of 107 prospective patients, the AIR score outperformed the modified Alvarado and the RIPASA scoring systems.

In the present study, it was seen that vomiting alone had a better area under ROC than nausea and vomiting combined. This shows the lack of sensitivity of the subjective symptom of nausea and the better discriminating ability of the objective parameter—vomiting. Anorexia—another subjective parameter—also had poor performance. Migratory pain, rebound tenderness, WBC count, and CRP value, all had an area under ROC above 0.7. Considering individual parameters, the CRP value was the best performing with an area under ROC of 0.789, followed by WBC count with an area under ROC of 0.762. The poorest performing among symptoms and signs was anorexia with an area under ROC of 0.37. The poorest performing laboratory investigation was shift to left with an area under ROC of 0.65. CRP was the best overall performing individual parameter in AIRS. WBC count was the best performing individual parameter in the Alvarado score. However, the combined scores of Alvarado and AIR score are better than individual parameters in suspected acute appendicitis.

This study has some limitations. First it is a relatively small size of the study population. Second is the cross-sectional nature of the study. Third, limitation is that only patients who underwent appendicectomy for suspected acute appendicitis were included. Fourth is the observational nature of the study. A prospective trial including patients with suspected acute appendicitis, evaluating the treatment decisions based on AIR score is required in the future. Despite these limitations, it is seen from this study and available literature that the AIR score outperforms the Alvarado score as a clinical prediction rule in patients with suspected acute appendicitis with higher specificity. The score is also seen to correlate with the severity of acute appendicitis. Use of AIR score may thus decrease the rate of negative appendicectomies. The AIR score is especially useful in situations where imaging is inconclusive and also in resource-limited areas where radiological imaging is not available.

## Conclusion

The AIR score which is a recently developed score outperforms the Alvarado score. The ease of application of the score, the sound construction, grading of parameters, avoiding of subjective parameters, and the introduction of CRP into scoring all make the appendicitis inflammatory response score into an attractive clinical prediction rule with higher specificity. The use of AIR score in resource-limited settings can decrease the rate of negative appendicectomy.
